# Probiotics as Means of Diseases Control in Aquaculture, a Review of Current Knowledge and Future Perspectives

**DOI:** 10.3389/fmicb.2018.02429

**Published:** 2018-10-12

**Authors:** Seyed Hossein Hoseinifar, Yun-Zhang Sun, Anran Wang, Zhigang Zhou

**Affiliations:** ^1^Department of Fisheries, Faculty of Fisheries and Environmental Sciences, Gorgan University of Agricultural Sciences and Natural Resources, Gorgan, Iran; ^2^Key Laboratory of Healthy Mariculture for the East China Sea, Ministry of Agriculture, Fisheries College, Jimei University, Xiamen, China; ^3^Key Laboratory for Feed Biotechnology of the Ministry of Agriculture, Feed Research Institute, Chinese Academy of Agricultural Sciences, Beijing, China

**Keywords:** disease control, immune responses, probiotics, fish, shellfish

## Abstract

Along with the intensification of culture systems to meet the increasing global demands, there was an elevated risk for diseases outbreak and substantial loss for farmers. In view of several drawbacks caused by prophylactic administration of antibiotics, strict regulations have been established to ban or minimize their application in aquaculture. As an alternative to antibiotics, dietary administration of feed additives has received increasing attention during the past three decades. Probiotics, prebiotics, synbiotics and medicinal plants were among the most promising feed supplements for control or treatments of bacterial, viral and parasitic diseases of fish and shellfish. The present review summarizes and discusses the topic of potential application of probiotics as a means of disease control with comprehensive look at the available literature. The possible mode of action of probiotics (Strengthening immune response, competition for binding sites, production of antibacterial substances, and competition for nutrients) in providing protection against diseases is described. Besides, we have classified different pathogens and separately described the effects of probiotics as protective strategy. Furthermore, we have addressed the gaps of existing knowledge as well as the topics that merit further investigations. Overall, the present review paper revealed potential of different probiont to be used as protective agent against various pathogens.

## The interactions between probiotics and diseases of fish and shellfish

### Probiotics: definition and history

Nowadays, several types of beneficial feed additive such as probiotics, prebiotics, and synbiotics are being used in aquaculture to improve growth performance, immune responses and disease resistance as well as an alternative to antibiotics (Irianto and Austin, 2002; Hoseinifar et al., 2016, 2017b; Sayes et al., 2018). The term “probiotics” arose from the Greek words “pro” and “bios” meaning “for life”; generally referred to microbial feed additives which confer host organism through modulation of intestinal microbiota. Parker (1974) was the first who defined probiotics as organisms and substances that affect microbial in intestine. According to the Food and Agriculture Organization (FAO) and the World Health Organization (WHO), probiotics are live microorganisms which are used orally having some tangible health benefits to the host (Hotel and Córdoba, 2001). Considering the difference between environment in aquatic ecosystem and those terrestrial animals, a modified definition proposed for probiotics in aquaculture by Merrifield et al. (2010b) as, “a probiotic organism can be regarded as a live, dead or component of a microbial cell, which is administered via the feed or to the rearing water, benefiting the host by improving disease resistance, health status, growth performance, feed utilization, stress response or general vigor, which is achieved at least in part via improving the hosts microbial balance or the microbial balance of the ambient environment.” The probiotics include different kinds of bacteria, bacteriophages, microalgae and yeast which have been widely used in aquaculture via water routine or feed supplement (Llewellyn et al., 2014) Currently, there are lots of commercially available probiotics in for of mono or multi-strains (Van Doan et al., 2017).

### Mode of actions on disease resistance

The extensive literature on probiotics revealed beneficial effects on host's gut defenses which has vital importance in diseases prevention as well digestive tract inflammation treatment (Azimirad et al., 2016; Modanloo et al., 2017). Apart from immunomodulation, probioticmicroorganisms, such as lactic acid bacteria, *Brevibacillus brevis, Vagococcus fluvialis*, and *Vibrio harveyi* (Lazado et al., 2011; Sugimura et al., 2011; Korkea-aho et al., 2012; Mahdhi et al., 2012; Sorroza et al., 2012), stick with the mucosal epithelium of gastrointestinal tract and help to resist pathogens (Luis-Villaseñor et al., 2011). In another way, probiotics increase feed digestibility through elevation of different digestive enzymes such as alginate lyases, amylases, and proteases (Zokaeifar et al., 2012). They also produce organic acids, fatty acids, biotin and vitamin B12, hydrogen peroxide, antibiotics, bacteriocins, siderophores, lysozyme (Sugita et al., 1991, 1992; Yan et al., 2002; Vine et al., 2006), which have positive effects on host health. Numerous studies have demonstrated that probiotics caused health benefits in aquatic organisms, such as Japanese flounder (Heo et al., 2013), black tiger prawns, *Penaeus monodon* (Rengpipat et al., 1998), whiteleg prawns (Chiu et al., 2007), and western king prawns (Hai et al., 2010).

#### Modulation of immune parameters

The first defense line against infections is innate immune responses (or non-specific immune responses) which include different cells and mechanisms that protect host organism from infectious diseases. It has been reported that probiotics can affect the elements of non-specific immune system such as mono-nuclear phagocytes (monocytes, macrophages) and polymorphonuclear leukocytes (neutrophils), natural killer (NK) cells etc. Previous studies revealed increment of leucocytes (Korkea-aho et al., 2012), monocytes (Aly et al., 2008b), erythrocytes, granulocytes, macrophage, and lymphocytes in various fishes following treatment with probiotics (Kim and Austin, 2006a,b; Nayak et al., 2007; Kumar et al., 2008). For instance, rainbow trout fed *Clostridium butyricum* showed increased resistance against vibriosis through affecting phagocytic activity of leukocytes (Sakai et al., 1995). Furthermore, dietary *Bacillus* sp. S11 positively affected cellular and humoral immunity in tiger shrimp (*Penaeus monodon*) which resulted in protection against disease (Rengpipat et al., 2000). Also, combined administration of *Bacillus* and *Vibrio* sp. in young white shrimp showed beneficial effects on growth performance, survival as well as resistance against *V. harveyi* and white spot syndrome virus (Antony et al., 2011). The authors attributed the protection to elevation of phagocytosis and antibacterial activity; indeed immunomodulation. Beside these results on shrimps, dietary *Lactobacillus rhamnosus* (ATCC 53103) (10^5^ CFU g^−1^) increased the respiratory burst in rainbow trout (*Oncorhynchus mykiss*)(Nikoskelainen et al., 2003). Therefore, probiotics are beneficial bacteria which not only capable of inhibiting pathogens, but also regulating the host immune system.Probiotics possess conserved microbe-associated molecular patterns (MAMPs), including peptidoglycan (PGN), lipoteichoic acids (LTA), S-layer protein A (SlpA), exopolysaccharides (EPS), flagellin and microbial nucleic acids which can be recognized by certain pattern recognition receptors (PRRs), and induces a signaling cascade that can result in the production of cytokines, chemokines, and other effector molecules thus activating the immune response in the host (Bron et al., 2012; Remus et al., 2012). During past years, there was increasing interests toward determination of mode of action of probiotics on intestinal immune system. In this regard, the researchers evaluated the possible relationship between TLR signaling-mediated recognition of probiotics and activation of the intestinal immunity. For example, it has been reported that TLR2 signaling pathway was involved in recognition of probiotic *Psychrobacter* sp. SE6 and inducing subsequently immune responses in grouper *Epinephelus coioides* (Sun et al., 2014).

#### Competition for binding sites

Competitive exclusion has been suggested as a mode of action of probiotic in prevention of pathogens (Mahdhi et al., 2012; Sorroza et al., 2012); achieved by colonization of probiotics in GI mucosal epithelium (Macey and Coyne, 2006; Merrifield et al., 2010a; Lazado et al., 2011; Korkea-aho et al., 2012). Different types of surface determinants suggested to be involved in probiotis interaction with intestinal epithelial cells and mucus which *per se* prevents pathogens colonization (so called competitive exclusion). The primary reason for this could be competitions for adhesion receptors (Montes and Pugh, 1993) which can antagonize pathogens (Luis-Villaseñor et al., 2011) and reduce their colonization (Chabrillón et al., 2005). This clearly shows the potential of probiotics administration as a substitute for antibiotics and other chemicals (Cheng et al., 2014). It has been reported that passive forces, electrostatic interactions, hydrophobic, steric forces, lipoteichoic acids were among the factors which affect adhesion of probiotics to attachment sites (Wilson et al., 2011). Westerdahl et al. (1991) stated that competition for attachment sites and nutrients following occupying mucosal surfaces could be possible mode of action for protective effects of probiotic against pathogens.

#### Production of antibacterial substances

In aquaculture, probiotics are used as an alternative to antibiotics and chemicals (Decamp et al., 2008; Van Hai et al., 2009; Heo et al., 2013). Though the mode of action through which probiotics exert antibacterial effects remained to be determined, many studies indicated that probiotics produced antibiotic compounds (Moriarty, 1998). Besides, reduce in pH following production of organic acids can inhibits growth of pathogenic bacteria (Ma et al., 2009). For example, Ramesh et al. (2015) reported antibacterial activity of *Bacillus licheniformis* and *B. pumilus*; which resist low pH and high bile concentrations. Another study with *Bacillus licheniformis* CPQB, revealed inhibition of *Vibrio alginolyticus* in whiteleg prawns (Ferreira et al., 2015). It has been demonstrated that *Lactobacillus* spp. (common probiotics) produce short chain fatty acids, diacetyl, hydro peroxide, and bactericidal proteins (Rengpipat et al., 1998; Verschuere et al., 2000; Faramarzi et al., 2011), which pre se improve immune responses as well as disease resistance (Raa, 1996; Gram et al., 1999). Consequently, probiotics can protect aquatic animals from challenge with pathogens by producing antibiotic compounds.

#### Competition for nutrients

The competition of nutrients has been considered among the mechanisms through which probiotics inhibit pathogens (Ringø et al., 2016). Previous study has reported that competition for iron is an essential element in marine bacteria (Verschuere et al., 2000). The majority of bacteria need Iron for their growth. However, there is limited available of iron in the tissues and body fluids of animals (Verschuere et al., 2000). The siderophores which are iron-binding agents, help bacteria to obtain the necessary amount of Iron for their growth. There is direct relation between production of siderophore and virulence of some pathogens (Gram et al., 1999).

The beneficial effects of Gram-positive genus *Bacillus* on water quality in culture environment has been reported in previous studies (Rafiee and Saad, 2005; El-Haroun et al., 2006; Hai, 2015; Dawood and Koshio, 2016). It seems that genus *Bacillus* is more effectual for converting organic matter to CO_2_ as well as balancing phytoplankton production (Balcázar et al., 2006). It has been reported that supplemented *F. vannamei* feed with *Bacillus* sp., *Saccharomyces cerevisiae, Nitrosomonas* sp., and *Nitrobacter* sp. (a commercial product) could decrease the concent rations of inorganic nitrogen and phosphate from 3.74 to 1.79 mg/L and 0.1105 to 0.0364 mg/L, respectively (Li et al., 2006).

In addition, probiotics also enhanced growth performance and feed utilization in aquatic animals through increasing digestive enzymes activity (Yu et al., 2009; Zokaeifar et al., 2012; Hoseinifar et al., 2017a). For example, Van Hai et al. reported that dietary probiotics (*Pseudomonas aeruginosa* and *Ps. Synxantha*) enhanced western king prawn growth performance (Van Hai et al., 2009; Hai et al., 2010). Recently research by Faturrahman et al. also revealed dietary probiotic (*Vibrio* Alg3.1Rf^R^-Abn1.2Rf^R^-enriched protein) improved growth rate of *Haliotis asinine* (Rohyati, 2015). The incease of digestive enzyme activity and improvement of the digestive process following treatment with probiotic has been attributed to production of extracellular enzymes such as proteases, carbohydrolases and lipases (Arellano-Carbajal and Olmos-Soto, 2002; Leonel Ochoa-Solano and Olmos-Soto, 2006; Soleimani et al., 2012; Eshaghzadeh et al., 2015; Hoseinifar et al., 2015a,b). Furthermore, considering provision of vital nutrients like fatty acids, biotin and vitamins, probiotics might be a complementary food source (Verschuere et al., 2000).

## Probiotics and bacterial diseases in fish (Table 1)

### Gram-positive bacteria

#### Lactic acid bacteria

Lactic acid bacteria (LAB) Gram positive, usually non-motile and non-sporing bacteria which mainly produce lactic acid during fermentation (Stanier et al., 1975). They were among the mostly studied probiotics (Merrifield et al., 2014). The extensive available literature revealed beneficial effects of LABs as probiotic on growth performance, immune responses and disease resistance shellfish (Ringø et al., 2010; Merrifield et al., 2014). Another important feature of these probiont strains is disease protection which has been reviewed in this section.

**Table 1:** Overview of the effects of probiotics against pathogenic bacteria in fish.

**Probiotic**	**Pathogen or disease**	**Fish species**	**Beneficial effects**	**Reference**
**GRAM-POSITIVE BACTERIA**				
*Carnobacteria. inhibens*	*A. salmonicida, Vibio ordalii, Yersinia ruckeri*	Atlantic salmon, Rainbow trout	Reduced mortalities	Robertson et al., 2000
*Carnobacterium divergens*	*V. anguillarum*	Atlantic cod	Reduced vibriosis	Gildberg et al., 1997
*Lactobacillus acidophilus*	*Pseudomonas fluorescens, Streptococcus iniae*	Nile tilapia	Improve immune function and disease resistance	Aly et al., 2008a
	*Staphylococcus xylosus, Aeromonas hydrophila* and *Streptococcus agalactiae*	African catfish	Reduced mortalities	Al-Dohail et al., 2011
*Lactobacillus rhamnosus*	*A. salmonicida*	Rainbow trout	Reduced mortalities	Nikoskelainen et al., 2001
	*Edwardsiella tarda*	Tilapia	Reduced mortalities	Pirarat et al., 2006
*Lactobacillus plantarum*	*Lactococcus* (*Lc*.) *garvieae*	Rainbow trout	Reduced mortalities	Vendrell et al., 2008
*Lactobacillus sakei*	*Edwardsiella tarda*	Rock bream	A non-significant decrease in the cumulative mortality	Harikrishnan et al., 2011
*Lactobacillus pentosus*	*Edwardsiella tarda*.	Japanese eel	Improved immune response and survival rate	Lee et al., 2013
*Lactobacillus brevis*	*A. hydrophila*	Tilapia	Significantly lower mortality	Liu et al., 2013
*Lactococcus lactis*	*Streptococcus iniae*	Olive flounder	Activated the innate immune system and protection against *pathogen* infection	Kim et al., 2013
	*Streptococcus iniae*	Olive flounder	Increased survival rate	Heo et al., 2013
*Leuconostoc mesenteroides*	furunculosis	Rainbow trout	Enhanced the immune response and disease resistance	Balcázar et al., 2007
	*Aeromonas salmonicida*	Brown trout	Enhanced the immune response and disease resistance	Balcázar et al., 2009
*Pediococcus pentosaceus*	*V. anguillarum*	Grouper	Significantly decreased the cumulative mortality	Huang et al., 2014
*Pediococcus acidilactici*	vertebral column compression syndrome (VCCS)	Rainbow trout fry	Increased survival rate	Aubin et al., 2005
*Enterococcus casseliflavus*	*Streptococcus iniae*	Rainbow trout	Improve growth performance and enhance disease resistance	Safari et al., 2016
*Enterococcus faecium*	*Edwardsiella tarda*	European eel	Reduced edwardsiellosis	Chang and Liu, 2002
*Enterococcus gallinarum*	*Vibrio anguillarum*	Sea bass	A moderated protective effect	Sorroza et al., 2013
*Vagococcus fluvialis*	*Vibrio anguillarum*	Sea bass	increased survival rate	Sorroza et al., 2012
*Bacillus subtilis* and *Bacillus licheniformis* (BioPlus2B)	*Y. ruckeri*	Trout	Increased survival rate	Raida et al., 2003
*Bacillus subtilis*	*A. hydrophila*	Indian major carp	Control of infection	Kumar et al., 2006
	*Aeromonas*	Rainbow trout	Increased survival rate	Newaj-Fyzul et al., 2007
	*Edwardsiella ictaluri*	Channel catfish and striped catfish	Reduced mortalities	Ran et al., 2012
	*Streptococcus* sp.	Grouper	Enhance the relative survival percentages	Liu et al., 2012
	*Streptococcus agalactiae*	Red hybrid tilapia	Reduced mortalities	Ng et al., 2014
*Bacillus pumilus*	*A. hydrophila*.	Tilapia	Enhance immune and health status and improve disease resistance	Aly et al., 2008b
*Bacillus circulans*	*A. hydrophila*	Catla catla	Enhanced the immune response and therefore survival	Bandyopadhyay and Das Mohapatra, 2009
*Bacillus subtilis Bacillus licheniformis*	*Streptoccocus iniae*	Olive flounder	Significantly higher survival ratio	Cha et al., 2013
*B. licheniformis*	*Streptococcus iniae*	Tilapia	Improved the disease resistance	Han et al., 2015
*Bacillus amyloliquefaciens*	*Yersinia ruckeri or Clostridium perfringens type D*	Nile tilapia	Better relative survival percentages	Selim and Reda, 2015
*Clostridium butyricum*	vibriosis	Rainbow trout	Enhance disease resistance	Sakai et al., 1995
	vibriosis	Chinese drum	Enhanced the phagocytic activity of leucocytes and therefore disease resistance to vibriosis	Pan et al., 2008b
*Micrococcus luteus*	*A. salmonicida*	Rainbow trout	Better survival	Irianto and Austin, 2002
	*A. hydrophila*.	Nile tilapia	Reduction in mortalities	Abd El-Rhman et al., 2009
*Rhodococcus* sp.	*V. anguillarum*	Rainbow trout	Significantly better protection	Sharifuzzaman et al., 2011
*Brochothrix thermosphacta*	*A. bestiarum*	Rainbow trout	Protected against skin infections	Pieters et al., 2008
*Kocuria* sp.	*V. anguillarum* and *V. ordalii*	Rainbow trout	Reduction in mortalities	Sharifuzzaman and Austin, 2010
**GRAM-NEGATIVE BACTERIA**				
*Pseudomonas fluorescens*	*V. anguillarum*	Rainbow trout	Reduced mortalities	Gram et al., 1999
*Pseudomonas chlororaphis*	*Aeromonas sobria*	Perch	Control *Aeromonas sobria* infection	Gobeli et al., 2009
*Pseudomonas* sp.	*F. psychrophilum*	Rainbow trout	Reduced mortalities	Korkea-aho et al., 2011
*Pseudomonas aeruginosa*	*A. hydrophila*	Rohu	Significantly higher post-challenge survival rates	Giri et al., 2012
	*Vibrio parahaemolyticus*	Zebrafish	PROTECT fish by inhibiting biofilm formation and enhancing defense mechanisms	Vinoj et al., 2015
*Aeromonas hydrophila*	*Aeromonas salmonicida*	Rainbow trout	Reduce infections	Irianto and Austin, 2002
	*Aeromonas salmonicida*	Goldfish	Controls infection	Irianto et al., 2003
*Aeromonas sobria*	*Lactococcus garvieae* and *Streptococcus iniae*	Rainbow trout	Improve the disease resistance	
*Aeromonas sobria*	*Aeromonas bestiarum*	Rainbow trout	Protected rainbow trout against challenge	Pieters et al., 2008
*Aeromonas veronii*	*A. hydrophila*	Common carp	Enhance disease resistance	Chi et al., 2014
*Shewanella putrefaciens*	*Vibrio anguillarum*	Gilthead seabream	Reduced mortalities	Chabrillón et al., 2006
	*Photobacterium damselae* sub sp. Piscicida	Senegalese sole	Improved survival	Diaz-Rosales et al., 2009
	*Photobacterium damselae* sub sp. Piscicida	Senegalese sole	Improved growth disease resistance	De la Banda et al., 2012
*Shewanella xiamenensis*	*A. hydrophila*	Grass carp	Improved disease resistance	Wu et al., 2015
*Enterobacter cloacae*	*Yersinia ruckeri*	Rainbow trout	High survival ratio	Capkin and Altinok, 2006
*Enterobacter amnigenus Enterobacter* sp.	*Flavobacterium psychrophilum*	Rainbow trout	Enhanced resistance to infection	Burbank et al., 2011
*Enterococcus faecalis*	*Aeromonas salmonicida*	Rainbow trout	Lower cumulative mortality	Rodríguez-Estrada et al., 2013
*Roseobacter sp*.	*V. anguillarum*	Turbot	Controlled *V. anguillarum* infection	Planas et al., 2006
*Phaeobacter* (*Roseobacter*) *gallaeciensis*	*V. anguillarum*	Cod larvae	Reduced the mortality by approximately 10%	D'Alvise et al., 2012
*Roseobacter* sp.	*Vibrio anguillarum*	Turbot	Significant decrease in cumulative mortality	Hjelm et al., 2004
*Vibrio alginolyticus*	*A. salmonicida*	Atlantic salmon	Significant decrease in cumulative mortality	Austin et al., 1995
*Zooshikella* sp.	*Streptococcus inane*	Olive flounder	Improve the innate immune response and control *streptococcus inane* infections	Kim et al., 2010
*Flavobacterium sasangense*	*A. hydrophila*	Common carp	Enhance immune response and disease resistance	Chi et al., 2014
**YEAST**				
*Saccharomyces cerevisiae*	*Aeromonas hydrophila*	Tilapia	Reduced mortality	Abdel-Tawwab et al., 2008
*Saccharomyces cerevisiae* var. *boulardii*	*Y. ruckeri*.	Rainbow trout	Improved disease resistance	Quentel et al., 2005
*Debaryomyces hansenii*	*Aeromonas hydrophila*	Leopard grouper	Improved immune function and disease resistance	Reyes-Becerril et al., 2011

##### Carnobacteria

*Carnobacteria* have been frequently isolated from fish intestine (Merrifield et al., 2014). It has shown antagonistic activity against different kinds of fish pathogens (Ringø et al., 2010). The *C. inhibens* K1 isolated from Atlantic salmon (*Salmo salar* L.) digestive tract inhibited fish pathogens under *in vitro* condition (Jöborn et al., 1997), and subsequently study showed that dietary administration of 5 × 10^7^ cells g^−1^ *C. inhibens* K1 for 14 days reduced mortalities caused by *A. salmonicida, Vibrio ordalii* and *Yersinia ruckeri* in Atlantic salmon and rainbow trout (Robertson et al., 2000). The *C. divergens* strain 6251, isolated from Artic charr (*Salvelinus alpinus* L.) foregut, showed growth-inhibitory effects against both *Aeromonas salmonicida* and *Vibrio anguillarum in vitro* (Ringø et al., 2002; Ringø, 2008). Also, dietary administration of *C. divergens* for 3 weeks reduced vibriosis caused by *V. anguillarum* in Atlantic cod (*G. morhua)* fry (Gildberg et al., 1997). Kim and Austin (2006a) characterized two *Carnobacteria* isolates obtained from rainbow trout intestine (*C. maltaromaticum* B26 and *C. divergens* B33). Both strains stimulated non-specific immunity and demonstrated effectiveness against *A. salmonicida* and *Y. ruckeri in vitro*. Løvmo Martinsen et al. (2011) reported that *C. maltaromaticum* which was previously isolated from Atlantic cod hindgut chamber could, to a certain extent, outcompete *V. anguillarum* in an unidentified mechanism.

##### Lactobacillus

The application of probiotic *Lactobacillus* spp. in fish aquaculture has been extensively studied (Merrifield et al., 2010a; Merrifield and Carnevali, 2014). *Lactobacillus* (*Lb*.) *acidophilus* improved immune responses and resistance against *Pseudomonas fluorescens* and *Streptococcus iniae* in Nile tilapia (Aly et al., 2008a,b). Similarly, African catfish (*Clarias gariepinus*) juveniles were fed *Lb. acidophilus* (3 × 10^7^ CFU g^−1^) for 12 weeks (Al-Dohail et al., 2011) and resistance against *Staphylococcus xylosus, Aeromonas hydrophila* gr2 and *Streptococcus agalactiae* (2 × 10^6^ CFU ml^−1^ intraperitoneal injection) were tested which revealed elevated resistance. Likewise, feeding rainbow trout with *Lb. rhamnosus* ATCC 53101 (10^9^ cells g^−1^) for 51 days resulting in a reduction of mortalities by *A. salmonicida* from ~ 53% to ~ 19% (Nikoskelainen et al., 2001). Furthermore, dietary supplemented with 10^8^ CFU g^−1^ and 10^10^ CFU g^−1^ *Lb. rhamnosus* for 14 days protected tilapia (*Oreochromis niloticus*) from acute septicemic death by experimental *Edwardsiella tarda* infection (Pirarat et al., 2011). Rainbow trout fed *Lb. plantarum* CLFP 238 at 10^7^ CFU g^−1^ of feed for 30 days showed a dramatic reduction in mortalities when challenged with pathogenic *Lactococcus* (*Lc*.) *garvieae* (Vendrell et al., 2008). Salinas et al. (2008) reported that *Lactobacillus delbrueckii* sp. *lactis* (CECT 287, Valencia, Spain) prevented *A. salmonicida* damaging effects in the foregut of Atlantic salmon. Likewise, a study on Gilthead seabream revealed *Lb. rhamnosus* and *Bifidobacterium lactis* notably reduced colonization of the pathogenic bacteria (*V. anguillarum, Photobacterium damselae* ssp. *piscicida, V. alginolyticus*, and *Vibrio harveyi*) (Chabrillon et al., 2006). It was also observed that *L. plantarum*, isolated from rainbow trout intestinal mucosa, could upregulate immune related genes expression and increase resistance against *Lc. garvieae* (Pérez-Sánchez et al., 2011). Feeding rock bream (*Oplegnathus fasciatus*) with *Lb. sakei* BK19 (2.2 × 10^7^ CFU g^−1^) resulted in non-significant decrease of mortality after challenge with *Edwardsiella tarda* (Harikrishnan et al., 2011). Also, *Lb. pentosus* PL11 improved immune responses as well as resistance of Japanese eel (*Anguilla* japonica) against *Edwardsiella tarda* (Lee et al., 2013). To test the protective effects of dietary supplementation of highly adhesive *Lactobacillus brevis* JCM 1170 (HALB) and less-adhesive *Lb. acidophilus* JCM 1132 (LALB) against the tilapia pathogen, *A. hydrophila* NJ-1, fish were immersed in strain NJ-1 for 14 days without supplemented feed. The results showed that diet containing 10^9^ cells g^−1^ of strain HALB/g feed (B3) showed significantly lower mortality (Liu et al., 2013). Recently, Beck et al. (2015) evaluated the mixture or single application of two host associated probiotics include *Lc. lactis* BFE920 isolated from bean sprout and *Lb. plantarum* FGL0001 isolated from olive flounder (*Paralichthys olivaceus*) hindgut, in olive flounder. After challenge with *Streptococcus iniae* (log_10_ 6.0 CFU/fish), the survival rate in the groups fed mixed probiotics and *Lb. plantarum* FGL0001, and the control were 55, 45, 35, and 20%, respectively. In a comparative view, it seems that *Lb. plantarum* was the most efficient *Lactobacillus* species in terms of disease bio-control. As the -aforementioned studies revealed, this species substantially improved resistance against various pathogenic bacteria. Besides disease protection, the species showed beneficial effects on growth performance and immune parameters (Van Doan et al., 2017). Hence, *Lb. plantarum* can be suggested as a promising tool for disease control in aquaculture.

##### Lactococcus

Balcázar et al. (2007) isolated *Lc. lactis* subsp. *lactis* (CLFP 100) and *Lc. lactis* subsp. *cremoris* (CLFP 102) from rainbow trout intestine. Subsequently, in a separate study, they administered *Lc. Lactis* in rainbow trout diet and observed increased immune parameters as well as protection against furunculosis (Balcázar et al., 2007). The same results observed with brown trout (*Salmo trutta*) challenged with *Aeromonas salmonicida* (Balcázar et al., 2009). Kim et al. (2013) reported that *Lc. lactis* BFE920 inhibits the growth of different pathogens including *Streptococcus iniae, S. parauberis, Enterococcus viikkiensis* as well as *Lactococcus garviae* under *in vitro* condition. The same authors supplemented olive flounder (*Paralichthys olivaceus*) diet with *Lc. lactis* BFE920 and after 2 weeks feeding observed activated the innate immune system which resulted in protection against *S. iniae* infection in both in experimental condition and large scale field condition. In accordance, Heo et al. (2013) reported that dietary *Lc. lactis* (10^8^ CFU g^−1^) elevated serum immune parameters (e.g., lysozyme, antiprotease, serum peroxidase, and blood respiratory burst activities) as well as resistance against *S. iniae* in olive flounder. Recently, Beck et al. (2015), in an study with olive flounder, observed that dietary administration of mixed probiotic *Lb. plantarum* FGL0001 and *Lc. lactis* BFE920, or single *Lc. lactis* BFE920 for 30 days could improve the survival rates after challenged with *S. iniae*. An overview of different *Lactococcus* spp. revealed that the main focus was on *Lc. Lactis* and this species was capable of protecting different fish species against bacterial pathogens.

##### Leuconostoc

Balcázar et al. (2007) reported that *Lc. mesenteroides* isolated from rainbow trout intestine inhibited the growth of various pathogens. The same research group supplemented rainbow trout and brown trout diets with *Lc. mesenteroides* (10^6^ CFU g^−1^) and observed immunomodulation and increased resistance against furunculosis (Balcázar et al., 2007) and *A. salmonicida* infection (Balcázar et al., 2009). Dietary application of *Lc. mesenteroides* CLFP 196 to rainbow trout at 10^7^ CFU g^−1^ of feed for 30 days dramatically reduced the mortalities following challenge with *L. garvieae* (Vendrell et al., 2008). However, *Lc. mesenteroides* subsp. *Mesenteroides*, obtained from rainbow trout intestine, failed to improve rainbow trout disease resistance to lactococcosis (Pérez-Sánchez et al., 2011). Although, there are limiting studies over *Leuconostoc* spp. potential to protect fish against diseases, but available results revealed beneficial effects of *Luc. mesenteroides*.

##### Pediococcus

Huang et al. (2014) isolated *P. pentosaceus* strain 4012 from cobia intestine and observed antagonistic effects on *V. anguillarum* under *in vitro* condition. Subsequently, dietary administration of *P. pentosaceus* 4012 significantly decreased the cumulative mortality of groupers after *V. anguillarum* infection (Huang et al., 2014). Dietary supplement with probiotic *P. acidilactici* increased resistance of rainbow trout fry against vertebral column compression syndrome (VCCS) (Aubin et al., 2005). Also, combined administration of galactooligosaccharides and *P. acidilactici* for 8 weeks improved the immune parameters and resistance against *S. iniae* in rainbow trout fingerlings. An overview of literature revealed increasing attentions toward administration of *P. acidilactici* as probiotic in aquaculture, recently. It seems that this species is capable to be considered as disease protection agent in aquaculture.

##### Enterococcus

Chang and Liu (2002) administered a commercial product containing *E. faecium* SF 68 in European eel, *Anguilla anguilla* diet and observed lower edwardsiellosis in fish exposed to *Edwardsiella tarda*. *E. gallinarum* showed a strong inhibitory effect against *V. anguillarum in vitro*, and under *in vivo* condition protected sea bass against *V. anguillarum* infection (Sorroza et al., 2013). Recently, Safari et al. (2016) evaluated the benefits of dietary administration of host-derived candidate probiotics *E. casseliflavus* in juvenile rainbow trout, and results showed that *E. casseliflavus* could improve growth performance and enhance disease resistance when challenged with *S. iniae*.

##### Vagococcus

Sorroza et al. (2012) supplemented sea bass diet with *Vagococcus fluvialis* (10^9^ cfu g^−1^) for 20 days and observed that probiotic fed fish had higher relative percent of survival (42.3%) than control group following challenge with *V. anguillarum*. This study showed the potential of *Vagococcus* spp. and highlighted the needs to additional research in future.

#### *Bacillus* sp.

*Bacillus* sp. as feed additives improves growth performance and immune response and disease resistance in fish has been extensively reviewed (Mingmongkolchai and Panbangred, 2018). Dietary administration of *B. subtilis* and *B. licheniformis* (BioPlus2B) improved trout resistance to infection with *Y. ruckeri* (Raida et al., 2003). Also, feeding Indian major carp, *Labeo rohita* with *B. subtilis* at 1.5 × 10^7^ CFU g^−1^ increased resistance against *A. hydrophila* infection (Kumar et al., 2006). Newaj-Fyzul et al. (2007) administered different forms (viable, formalized or sonicated cells or cell-free supernatant) of *B. subtilis* AB1 in rainbow trout diet and observed higher resistance against *Aeromonas* (Newaj-Fyzul et al., 2007). Furthermore, *B. subtilis* (8 × 10^7^ CFU g^−1^) reduced mortalities *Ictalurus punctatus* and striped catfish, *Pangasianodon hypophthalmus* following challenge with *Edwardsiella ictaluri* (Ran et al., 2012). Liu et al. (2012) proved that dietary *B. subtilis* (10^4^, 10^6^, and 10^8^ CFU g^−1^) for 14 and 28 days was able to enhance the relative survival percentages of grouper, *Epinephelus coioides* challenged with *Streptococcus* sp. A diet supplemented with 0.1 or 0.3% *B. subtilis* enhanced prophylactic property of red hybrid tilapia, *Oreochromis* sp. against pathogenic *Streptococcus agalactiae* (Ng et al., 2014).

Aly et al. (2008b) reported that feeding tilapia with 10^6^ and 10^12^ cells g^−1^ *B. pumilus* enhanced immune and health status and improve resistance against *A. hydrophila*. *B. pumilus* has also reported to dramatically improved survival of “Loco” *Concholepas concholepas* larvae (Leyton et al., 2012). Similarly, Sun et al. (2009) reported that *B. pumilus* SE5 and *B. clausii* DE5 obtained from orange-spotted grouper *Epinephelus coioides*, inhibited growth of pathogenic Staphylococcus *aureus, V. harveyi* and *V. parahaemolyticus* under *in vitro* condition. Also, feeding grouper *E. coioides* larvae with copepod (*P. annandalei*) enriched *B. clausii* DE5 and *B. pumilus* noticably larval survival (Sun et al., 2013).

Bandyopadhyay and Das Mohapatra (2009) isolated *Bacillus circulans* PB7 from *Catla catla* intestine, and subsequently added to *Catla catla* fingerlings diet at rate of 2 × 10^4^, 2 × 10^5^, or 2 × 10^6^ cells per 100 g. After 60 days feeding elevated immune parameters as well as resistance against *A. hydrophila* infection. Likewise, feeding Olive flounder (Paralichthys olivaceus) with (*B. subtilis, B. pumilus*, and *B. licheniformis*) at rate of 10^10^ CFU g^−1^ elevated resistance against *S. iniae* (Cha et al., 2013). Han et al. (2015) stated that feeding with commercial *B. licheniformis* improved the disease resistance against *Streptococcus iniae* infection in tilapia. Similarly, Nile tilapia fed with 1 × 10^6^ and 1 × 10^4^ CFU g^−1^ of *B. amyloliquefaciens* for 30 days showed higher resistance against pathogenic *Yersinia ruckeri* or *Clostridium perfringens* type D (Selim and Reda, 2015). Interestingly, intraperitoneally administration of cellular components (cell wall proteins and whole cell proteins) of *Bacillus licheniformis* and *B. pumilus* have been reported to improve immune parameters which *per se* protected rohu *Labeo rohita* (Hamilton) against *A. hydrophila* infection (Ramesh et al., 2015). The overview of literature regarding *Bacillus* spp. as probiotic aimed at elevation of disease resistance revealed more information on *B. subtilis*. The extensive research on this species revealed high potential for immunomodulation and disease protection. Indeed, *B. subtilis* can be considered as beneficial agent for disease bio-control.

#### Other gram-positive bacteria

##### Clostridium butyricum

Sakai et al. (1995) demonstrated that dietary *C. butyricum* increased rainbow trout protection vibriosis. Pan et al. (2008a) stated that *C. butyricum* CB2 showed strong antagonistic activity to pathogenic *A. hydrophila* and *V. anguillarum*. Subsequently, oral administration of live or dead *C. butyricum* CB2 at dose of 10^8^ CFU g^−1^ feed enhanced the phagocytic activity of leucocytes and resistance to vibriosis in Chinese drum, *Miichthys miiuy* (Basilewsky) (Pan et al., 2008b).

##### Micrococcus

Dietary application of probiotic *Micrococcus luteus* increased rainbow trout survival after *A. salmonicida* challenge (Irianto and Austin, 2002). Abd El-Rhman et al. (2009) reported that Nile tilapia fed *M. luteus* containing diets for 6-days per week for 90 days showed decreased mortality following *A. hydrophila* challenged.

##### Rhodococcus

It has reported that the cellular components (cell wall proteins and whole cell proteins) of *Rhodococcus* SM2 increased rainbow trout protection against *V. anguillarum* (Sharifuzzaman et al., 2011).

##### Brochothrix

Dietary administration of *Brochothrix thermosphacta* BA211 (10^10^ cells g^−1^) protected rainbow trout against skin infections challenged with *A. bestiarum*, i.e., mortalities reduced from 88 to 22%, however, the probiotic had no effect against ichthyophthiriasis (caused by the parasite *Ichthyophthirius multifiliis*) (Pieters et al., 2008).

##### Kocuria

Sharifuzzaman and Austin (2010) isolated *Kocuria* SM1 rainbow trout digestive tract and subsequently added to rainbow trout diet at rate of 10^8^ cells g^−1^. They observed higher protection against challenge with *V. anguillarum* and *V. ordalii*.

### Gram-negative bacteria

#### Pseudomonas

Rainbow trout exposed to *P. fluorescens* AH2 at rate of 10^5^ CFU/ml for 5 days showed lower mortality after *V. anguillarum* challenge (Gram et al., 1999), while the probiotic did not confer protection of salmon against furunculosis (Gram et al., 2001). *P. chlororaphis* strain JF3835, obtained from perch (*Perca fluviatilis* L.) intestine, has ability to control *Aeromonas sobria* infection in perch (Gobeli et al., 2009). *Pseudomonas* M162 showed *in vitro* inhibition to *Flavobacterium psychrophilum*, and dietary application of M162 increased rainbow trout resistance against *F. psychrophilum* infection (Korkea-aho et al., 2012). The same research group evaluated protection caused by various strains of *Pseudomonas* M174 in rainbow trout and observed highest protection against *F. psychrophilum* caused by M174 strain (Korkea-aho et al., 2011). Giri et al. (2012) fed Labeo rohita with 10^7^ and 10^9^ CFU g^−1^ P. aeruginosa VSG-2 and evaluated fish resistance against A. hydrophila. The results revealed that probiotic fed fish had significantly higher resistance against A. hydrophila infection (Giri et al., 2012). Similarly, oral administration of P. aeruginosa PsDAHP1 inhibited biofilm formation and increased defense mechanisms which *per se* elevated zebrafish protection from *V. parahaemolyticus* DAHV2 infection (Vinoj et al., 2015).

#### Aeromonas

Dietary administration of *A. hydrophila* has been reported to reduce mortality caused by *A. salmonicida* in rainbow trout (Irianto and Austin, 2002). Similarly, Irianto et al. (2003) showed that feeding with formalin-inactivated A. hydrophila A3-51 increased goldfish (Carassius auratus) resistance against A. salmonicida. Likewise, rainbow trout fed A. sobria GC2 at rate of 5 × 10^7^ cells g^−1^ showed improved resistance to *L. garvieae* and *S. iniae* (Brunt and Austin, 2005). Also, Pieters et al. (2008) demonstrated that feeding with 10^8^ cells g^−1^ *A*. sobria GC2 and 10^10^ cells g^−1^ Brochothrix thermosphacta BA211 improved rainbow trout resistance against causal agent of fin rot (i.e., *A. bestiarum*). Recently, Chi et al. (2014) isolated A. veronii BA-1 from common carp (Cyprinus carpio) intestine. Dietary administration of isolated strain (1 × 10^8^ cell g^−1^) beneficially affected immune parameters as well as resistance against A. hydrophila in carp.

#### Shewanella

Chabrillón et al. (2006) fed gilthead seabream (Sparus aurata) with S. putrefaciens (Pdp11) at rate of 10^8^ CFU g^−1^ and then challenged with V. anguillarum DC11R2a. The results revealed significantly lower mortality in probiotic fed fish (10%) compared to control group (56%). In a study with Senegalese sole (*Solea senegalensis*), Diaz-Rosales et al. (2009) evaluated probiotic potential of *S. putrefaciens* Pdp11 and *S. baltica* Pdp13. They observed elevated immune responses as well as resistance against *Photobacterium damselae* sub sp. Piscicida. Also, De la Banda et al. (2012) evaluated effectiveness of different forms (fresh and lyophilized cells) of *S. putrefaciens* Pdp11, in juvenile Senegalese sole diet and observed improved growth and protection against *P. damselae* subsp. *piscicida*. Recently, the same group reported that dietary *S. putrefaciens* Pdp11 modulated immune related genes expression, intestinal microbiota as well as intestinal conditions which *per se* improved stress tolerance caused by crowding condition. (Tapia-Paniagua et al., 2014). Dietary application of 1 × 10^8^ cell g^−1^ *S. xiamenensis* A-1 and *S. xiamenensis* A-2, which isolated from grass carp (*Ctenopharyngodon idellus*) intestine, for 28 days decrease the survival of grass carp after experimentally challenged with A. hydrophila (Wu et al., 2015).

#### Enterobacter

Capkin and Altinok (2006) isolated E. cloacae from rainbow digestive tract and supplemented trout diet with isolated strain at rate of 10^8^ CFU g^−1^. Interestingly, following challenge with Yersinia ruckeri, probiotic fed fish showed significantly higher survival (99.2%) compared those fed control diet (35%). Probiotic strains C6-6 and C6-8 which supposed to be *E. amnigenus* and *Enterobacter* sp., inhibited F. psychrophilum (Burbank et al., 2011). Moreover, supplementation of rainbow trout with 10^6^ to 10^8^ cells g^−1^ of those probiotic resulted in higher resistance to *Flavobacterium psychrophilum* infection (Burbank et al., 2011). Also, inclusion of inactivated E. faecalis in rainbow trout diet at rate of 5 g kg^−1^ decreased mortality caused by experimental A. salmonicida infection (Rodríguez-Estrada et al., 2013).

#### Roseobacter

It has been reported that rotifers enriched with *Roseobacter* 27-4 increased turbot, *Scophthalmus maximus* L., larvae protection against *V. anguillarum* infection (Planas et al., 2006). An isolate from seawater in scallop (*Pecten maximus*) identified as *Phaeobacter* (*Roseobacter*) *gallaeciensis* BS107 (DSM17395), which antagonized fish pathogenic bacteria *in vitro* and reduced the mortality by approximately 10% in cod larvae upon challenge with *V. anguillarum* (D'Alvise et al., 2012). Treatment of Turbot (*Scophthalmus maximus*) larvae with 10^7^ CFU mL^−1^ Roseobacter sp. strain 27-4, decreased cumulative mortality following challenge with V. anguillarum (Hjelm et al., 2004). Likewise, feeding turbot larvae with Roseobacter sp. strain 27-4 enriched rotifers improved protection against V. anguillarum, (Planas et al., 2006).

#### Vibrio

*Vibrio alginolyticus* showed *in vitro* inhibition to *V. ordalii, V. anguillarum, A. salmonicida* and *Y. ruckeri*, and *in vivo* protection to Atlantic salmon challenged with *A. salmonicida* (Austin et al., 1995). Dietary administration of V. fluvialis resulted in higher survival in rainbow trout challenged with *A. salmonicida* (Irianto and Austin, 2002).

#### Zooshikella

Kim et al. (2010) showed that different levels (3.4 × 10^4^, 3.5 × 10^6^ and 3.4 × 10^8^ CFU g^−1^) of dietary *Zooshikella* sp. JE-34 helps to improve the innate immune response and control streptococcus inane infections in olive flounder (Paralichthys olivaceus).

#### Flavobacterium

Chi et al. (2014) supplemented carp diet with Flavobacterium sasangense BA-3 (1 × 10^8^ cell g^−1^) isolated from the common carp intestine for 28 days. They observed enhanced immune parameters as well as resistance against A. hydrophila infection.

### Yeast

The potential of yeast as probiotic to improve disease resistance has been demonstrated in several studies. Abdel-Tawwab et al. (2008) reported that diets supplemented with baker's yeast *S. cerevisiae* at dose of 0.25, 0.50, 1.0, 2.0, or 5.0 g yeast/kg reduced mortality in tilapia after intraperitoneal injection pathogenic *A. hydrophila*. Subsequently, the same group observed that Baker's yeast improves the resistance against the water-borne Cu toxicity in Galilee tilapia *Sarotherodon galilaeus* (L.) (Abdel-Tawwab et al., 2010). Quentel et al. (2005) reported that singular or combined administration of *P. acidilactici* and *S. cerevisiae* var. *boulardii* improved rainbow trout resistance against *Y. ruckeri*. Reyes-Becerril et al. (2011) supplemented Leopard grouper (*Mycteroperca rosacea*) diet with *Debaryomyces hansenii* CBS 8339 (10^6^ CFU g^−1^) for 5 weeks. At the end of feeding trial, probiotic fed fish ad noticeably higher immunoglobulin M (IgM) level, catalase (CAT) and superoxide dismutase (SOD) activities following *A. hydrophila* AH-315 challenge. Generally, the majority of studies performed on yeasts revealed beneficial effects on immune system (Hai, 2015). Hence, it seems that they can be considered as beneficial means of disease control and control.

## Probiotics and bacterial diseases in shellfish (Table 2)

## Gram-positive bacteria

### Lactic acid bacteria

Ajitha et al. (2004) supplemented Indian white shrimp (*Penaeus indicus*) diet with as single dose (5 × 10^6^ CFU g^−1^) of different probiotics including *Lb. acidophilus, S. cremoris, Lb. bulgaricus* 56 or *L. bulgaricus*57 at doses of for 4 weeks and at the end of feeding trial shrimp exposed to experimental *Vibrio alginolyticus* infection. The results revealed substantially higher resistance (56–72%) compared control group (20%) (Ajitha et al., 2004). Also, dietary supplemented with 10^10^ CFU kg^−1^ of *Lb. plantarum* upregulated proPO and PE genes, enhanced PO and SOD activities as well as resistance against *V. alginolyticus* in white shrimp (Chiu et al., 2007). Similarly, Vieira et al. (2010) reported that diet supplemented with probiotic *Lb. plantarum* modulated intestinal microbiota as well as resistance against *V. harveyi*. In addition, a *Lactobacillus* sp. has been reported to improve survival by 72% and performance of pearl oyster, *P. mazatlanica* (Aguilar-Macias et al., 2010). Furthermore, in a study with juvenile tiger shrimp (*Penaeus monodon*) *Lb. acidophilus* 04 (10^5^ CFU g^−1^) was administered for 1 month and increased resistance (80% survival) was observed following exposure with pathogenic *V. alginolyticus* (Sivakumar et al., 2012). Interestingly, Dash et al. (2015) administered heat-killed form of *Lb. plantarum* at rate of 10^8^ CFU g^−1^ in *M. rosenbergii* diet for 90 days. While no significant effects were observed on growth performance, feeding on probiotic supplemented diet noticeably enhanced immune responses and disease resistance.

**Table 2:** Overview of the effects of probiotics against pathogenic bacteria in shellfish.

**Probiotic**	**Pathogen or disease**	**Shellfish species**	**Beneficial effects**	**Reference**
**GRAM-POSITIVE BACTERIA**				
*Lactobacillus acidophilus Streptococcus cremoris Lactobacillus bulgaricus*	*Vibrio alginolyticus*	Indian white shrimp (*Penaeus indicus*)	Higher survival rate	Ajitha et al., 2004
*Lactobacillus plantarum*	*Vibrio alginolyticus*	White shrimp (*Litopenaeus vannamei*)	Increased clearance efficiency to *V. alginolyticus* and the survival rate	Chiu et al., 2007
	*Vibrio harveyi*	White shrimp (*Litopenaeus vannamei*)	Enhanced disease resistance	Vieira et al., 2010
*Lactobacillus acidophilus*	*Vibrio alginolyticus*	Shrimp (*Penaeus monodon*)	Higher survival ratio	Sivakumar et al., 2012
*Enterococcus faecium Lactococcus garvieae*	*Vibrio harveyi* and *Vibrio parahaemolyticus*	Shrimp (*Penaeus monodon*)	Enhanced disease resistance	Swain et al., 2009
*Pediococcus acidilactici*	*Vibrio nigripulchritudo*	Blue shrimp (*Litopenaeus stylirostris*)	Higher survival rate	Castex et al., 2010
*Lactobacillus pentosus Enterococcus faecium*	*Vibrio parahaemolyticus*	Shrimp (*Litopenaeus vannamei*)	Enhanced the survival rate	Sha et al., 2016
*Bacillus subtilis*	*Vibrio harveyi*	Shrimp (*Penaeus monodon*)	Enhanced the survival rate	Vaseeharan and Ramasamy, 2003
	*Vibrio harveyi*	Shrimp (*Litopenaeus vannamei*)	Enhanced the survival rate	Balcázar et al., 2007; Zokaeifar et al., 2012; Liu et al., 2014
*Bacillus* sp.	*Vibrio harveyi*	Shrimp (*Penaeus monodon*)	Enhanced the survival rate	Rengpipat et al., 1998
*Streptococcus phocae*	*Vibrio harveyi*	Shrimp (*Penaeus monodon*)	Enhanced the survival rate	Swain et al., 2009
*Arthrobacter sp*.	*Vibrio parahaemolyticus*	Shrimp (*Litopenaeus vannamei*)	Significantly enhanced the immune parameters and significantly decreased mortalities	Li et al., 2008
**GRAM-NEGATIVE BACTERIA**				
*Streptomyces* sp.	*Vibrio harveyi*	Black tiger shrimp (*Penaeus monodon*)	Better survival and growth performance	Das et al., 2010
*Pseudomonas aeruginosa*	*Vibrio harveyi*	Western king prawns (*Penaeus latisulcatus*)	Improved the survival rate	Van Hai et al., 2009
*Pseudomonas sp*.	*Vibrio harveyi*	Whrimp (*Penaeus monodon*)	Improved the survival rate	Pai et al., 2010
*Alteromonas macleodii Neptunomonas sp*.	*Vibrio splendidus*	Greenshell mussel(*Perna canaliculus)*	Improved survival and suppress naturally occurring vibrios	Kesarcodi-Watson et al., 2010
	*Vibrio coralliilyticus* and *V. splendidus*	Scallop (*Pecten maximus*	Improved the survival rate	Kesarcodi-Watson et al., 2012
	*Vibrio coralliilyticus* and *Vibrio pectenicida*	Flat oyster (*Ostrea edulis*)	Improved the survival rate	Kesarcodi-Watson et al., 2012
*Phaeobacter gallaeciensis, Pseudoalteromonas*	*Vibrio coralliilyticus* and *V. splendidus*	Scallop (*Pecten maximus)*	Improved the survival rate	Kesarcodi-Watson et al., 2012
**YEAST**				
*Phaffia rhodozyma Saccharomyces cerevisiae*	vibriosis	Shrimp (*Litopenaeus vannamei*)	Improve resistance against vibriosis	Scholz et al., 1999

Swain et al. (2009) reported that feeding with *E. faecium* MC13 and *Lactococcus garvieae* B49 protected post larval shrimp, *P. monodon*, against challenge with *V. harveyi* and *V. parahaemolyticus*. Similarly, feeding blue shrimp (*Litopenaeus stylirostris*) with probiotic *P. acidilactici* enhanced protection against *V. nigripulchritudo* SFn1; the mortality in probiotic and control group were 25 and 41.7%, respectively (Castex et al., 2010). Dietary administration of *Lb. pentosus* HC-2 and *E. faecium* NRW-2 noticeably enhanced resistance against pathogenic *V. parahaemolyticus* ATCC 17802 in *L. vannamei* (Sha et al., 2016).

### Bacillus

To study protective effects of *Bacillus subtilis* BT23, Vaseeharan and Ramasamy (2003) treated black tiger shrimp with 10^6^-10^8^ CFU ml^−1^ probiotic for 6 days and then challenged with *V. harveyi*. The results revealed significantly lower mortality in treated groups (Vaseeharan and Ramasamy, 2003). Similarly, Balcázar et al. (2007) fed *L. vannamei* juvenile with *B. subtilis* for 28 days and then exposed to pathogenic *V. harveyi* for 24 h. The results revealed substantially lower mortality in treated group (18.25%) compared to those in control (51.75%) in the control group (Balcázar et al., 2007). Also, Zokaeifar et al. (2012) tested combined administration of two probiotic strains (*B. subtilis* L10 and G1) in juvenile white shrimp. Shrimps were fed with two levels of 10^5^ and 10^8^ CFU g^−1^ of selected probiotics for 8 weeks. At the end of feeding trial elevated growth performance, digestive enzyme activity, upregulated immune related genes as well as resistance against *V. harveyi* were observed (Zokaeifar et al., 2012). Liu et al. (2014) reported that dietary administration of *B. subtilis* strain S12 (isolated from *L. vannamei* digestive tract), beside *in vitro* antagonistic activity against aquatic animal pathogens, improved resistance against *V. harveyi* infection (Liu et al., 2014).

Rengpipat et al. (1998) reported that supplementation of black tiger shrimp with different forms (i.e., of fresh cells, fresh cells in normal saline solution and a lyophilized form) of *Bacillus* S11 for 100 days resulted in significantly higher growth performance and survival. Also, the authorsperformed experimental challenge *V. harveyi* at the end of feeding trial and surprisingly observed no mortality in probiotic fed shrimps, while survival rate was just 26% in control group (Rengpipat et al., 1998). Subsequently, the same research group studied possible effects of *Bacillu*s S11 and concluded limited improvement in resistance against *V. harveyi* (Rengpipat et al., 2003). In another routes of administration, Luis-Villaseñor et al. (2011) isolated four *Bacillus* strains from white shrimp digestive tract and added to white shrimp culture water at rate of 1 × 10^5^ CFU mL^−1^ daily. Thereafter, the authors observed elevated overall survival of *L. vannamei* larvae (Luis-Villaseñor et al., 2011). In another study with post larvae, Ravi et al. (2007) claimed elevated resistance against *V. harveyi* following treatment of post larvae with *Paenibacillus* sp. EF012164 and *Bacillus cereus* DQ915582 (Ravi et al., 2007). The same results were also reported in case of *Bacillus* sp. P11 which resulted in substantially higher survival in comparison with control group (0%) following experimental challenge with *V. harveyi* (Utiswannakul et al., 2011). The literature review denote that, perhaps, the most studied and effective probiont in shrimp culture is *B. subtilis*. This species showed positive effects on shrimp resistance to various pathogens. Hence, can be considered as a means of disease control and control in shrimp aquaculture.

### Other gram-positive bacteria

Swain et al. (2009) demonstrated that feeding *P. monodon* post larvae with Streptococcus *phocae* P180 significantly improved growth performance as well as protection against *V. harveyi*. However, the probiotic failed to protect the animals against *V. parahaemolyticus* (Swain et al., 2009). The probiotic *Arthrobacter* XE-7 was administered orally at four different doses of 0, 10^6^, 10^8^, and 10^10^ CFU g^−1^ feed for 63 days in Pacific white shrimp, *L. vannamei*. Li et al. (2008) supplemented shrimp diet with *Arthrobacter* XE-7 and observed beneficial effects on intestinal microbiota, immune response as well as resistance against *V. parahaemolyticus* (Li et al., 2008).

### Gram-negative bacteria

#### Vibrio

Thompson et al. (2010) demonstrate *in vitro* growth inhibition of shrimp pathogens by probiotic *V. gazogenes* NCIMB 2250. Also, the same author revealed that feeding white shrimp with dietary *V. gazogenes* NCIMB 2250 elevated performance and health status as well as decreased of *Vibrio* sp. count in intestinal microbiota (Thompson et al., 2010). Likewise, *Vibrio* NE17 isolated from the egg samples improved performance as well as immune parameters of freshwater prawn, *Macrobrachium rosenbergii* (Mujeeb Rahiman et al., 2010). Also, a abalone, *H. rufescens* revealed that combined administration of three probiotics (*Vib*rio sp. C21-UMA, Agarivorans albus F1-UMA and Vibrio sp. F15-UMA) using macroalgae *M. integrifolia* as vector increased significantly the survival of, in a period of 210 days (Silva-Aciares et al., 2011).

#### Streptomyces

In 2016, Tan et al. (2016) have reviewed the use of the genus Streptomyces bacteria as a probiotic in controlling diseases and improving the health and quality of aquaculture production. Das et al. (2010) used Marine *Strepto*myces strains (CLS-28, CLS-39) in Artemia culture and concluded that this probiotic significantly increased resistance of *Artemia* nauplii and adult against *V. harveyi* and *V. proteolyticus* (Das et al., 2010). Thereafter, they supplemented black tiger shrimp post larvae diet with 1% *Strepto*myces for 15 days. The results revealed improved resistance against *V. harveyi* and growth performance in probiotic fed shrimps (Das et al., 2010).

#### Pseudomonas

Van Hai et al. (2009) supplemented western king prawns (Penaeus latisulcatus) diet with a single dose (20 × 10^5^ CFU kg^−1^) of P. aeruginosa and P. synxantha for 84 days and reported higher survival rate in P.aeruginosa fed group. Also, combined administration of those probiotic was more effective than singular. Pai et al. (2010) reported *in vitro* inhibition of V. harveyi MCCB 111 growth by Pseudomonas MCCB 102 and MCCB 103. Also, *in vivo* study revealed noticeable increase of tiger shrimp larvae survival against V. harveyi MCCB 111.

#### Alteromonas

*Alteromonas macleodii* 0444 has been reported to control of *Vibrio splendidus* infection in Greenshell mussel, *Perna canaliculus*, which *per se* caused increase in survival rate and natural Vibrios in the culture environment (Kesarcodi-Watson et al., 2010). Also, the same research group showed that *A. macleodii* 0444 protected scallop (*Pecten maximus*) and flat oyster (*Ostrea edulis*) larvae against *V. coralliilyticus* and *V. splendidus, V. pectenicida* infections (Kesarcodi-Watson et al., 2012).

#### Neptunomonas

Kesarcodi-Watson et al. (2010) demonstrated that *Neptunomonas* 0536 was capable of controlling infection caused by *V. splendidus* in Greenshell mussel (*P. canaliculus*). Also, the same research group highlighted the potential of this probiotic to protect scallop and flat oyster from larvae against *V. coralliilyticus, V. splendidus* and *V. pectenicida* (Kesarcodi-Watson et al., 2010, 2012).

#### Phaeobacter

*Phaeobacter gallaeciensis* protected scallop larvae against *V. coralliilyticus* and *V. splendidus*. Also, the same probiotic strain protected flat oyster larvae against *V. coralliilyticus* and *V. pectenicida*, and Pacific oyster larvae against *V. coralliilyticus* but not *V. pectenicida* (Kesarcodi-Watson et al., 2012).

#### Pseudoalteromonas

Kesarcodi-Watson et al. (2012) reported that *Pseudoalteromonas* D41 as probiotic increased resistance of scallop larvae and Pacific oysters against *V. splendidus* and *V. coralliilyticus*, respectively.

### Yeast

To the best of our knowledge there is limited information regarding application of yeasts as probiotic in shellfish aquaculture. In an early study Scholz et al. (1999) supplemented white shrimp with 1% *Phaffia rhodozyma* and *S. cerevisiae* and reported elevation of protection against vibriosis. Furthermore, feeding pearl oyster, *P. mazatlanica* with marine yeast (*Yarrowia lipolytica*) enriched microalgae resulted in enhanced growth and survival (Aguilar-Macias et al., 2010).

## Probiotics and viral diseases in fish

The occurrence of viral diseases causes mass mortality in aquaculture practice and considering still there is limited effective vaccine this could a bottleneck for aquaculture industry which resulted in substantial economic loss. In this regard, the potential of probiotics to be used as a means of controlling viral disease has been shown in few studies. For instance, Balcázar et al. (2007) in an *in vitro* study demonstrated antiviral activity of probiotic strains (including *Vibrios* spp., *Pseudomonas* spp., *Aeromonas* spp.) against *infectious hematopoietic necrosis virus* (*IHNV*). Likewise, Maeda et al. (1997) reported that *Pseudoalteromonas undina*, VKM-124 improved larval survival by giving the larvae a protection against *Sima-aji Neuro Necrosis Virus* (*SJNNV*) when added to Yellow Jack (*Carangoides bartholomaei*) larval tanks. Harikrishnan et al. (2010) studied antiviral activity of dietary two commercial probiotics (Lactobacil and/or Sporolac) in Olive flounder. The results revealed that both probiotics increased fish resistance against lymphocystis disease virus (LCDV) infection (Harikrishnan et al., 2010). The possible control of iridovirus in grouper (*Epinephelus coioides*) through dietary administration of probiotics (*Lb. plantarum*) was studied by Son et al. (2009). The results revealed higher survival in probiotic fed fish compared control group. In another study Liu et al. (2012) tested possible protection of grouper against iridovirus using dietary *B. subtilis* E20 and observed 50% higher survival than those in non-probiotic group. Likewise, dietary *S. cerevisiae* at rate of 5.3 × 10^7^ CFU kg^−1^ protected grouper against iridovirus (GIV) infection (Chiu et al., 2010). Indeed, while fish fed control diet had 16.7% survival, probiotic fed fish survival was 43.3%. Although there are extensive literature regarding immunomodulatory effects of probiotics, they are not enough to speculate potential anti-viral effects of probiotics. Therefore, more studies should be conducted to illustrate the effect of probiotics on the viral diseases of fish and possible mechanisms.

## Probiotics and viral diseases in shellfish

Unlike fish, shrimp aquaculture suffers from substantial economical loses due to occurrence and spread of different viral diseases like white spot syndrome virus (WSSV), lymphocystis disease virus (LCDV), infectious hypodermal and hematopoietic necrosis virus (IHHNV) etc. Treatment of shrimp culture environment or feed with probiotics has been suggested as efficient means of prevention and controlling viral diseases (Lakshmi et al., 2013). For instance, *Vibrio* spp. obtained from tiger shrimp hatchery showed strong antagonistic activity against IHNV and *Oncorhynchus masou* virus (OMV) (Direkbusarakom et al., 1998). The majority of studies practiced dietary administration of probiotics and tested anti-viral effect in different shrimp species. Rodríguez et al. (2007) stated that treatment of *L. vannamei* with 10^5^ CFU mL^−1^ probiotic *V. alginolyticus* significantly increased resistance against WSSV compared to non-treated shrimps. Moreover, dietary administration of 10^10^ CFU g^−1^ *B. megaterium* has resulted in higher survival and increased protection against WSSV (Li et al., 2009). Also, Leyva-Madrigal et al. (2011) reported that feeding white shrimp with either *P. pentosaceus* or *Staphylococcus hemolyticus* decrease WSSV infection. On the contrary, dietary supplemented with 10^5^ CFU g^−1^ of a mixture lactic acid bacteria (BAL3, BAL7, BC1, and CIB1) had no significant effects on *L. vannamei* resistance against WSSV infections (Partida-Arangure et al., 2013). Recently, Chai et al. (2016) isolated *Bacillus* PC465 from *Fenneropenaeus chinensis* gut and evaluated its anti-viral effects via dietary administration. The results showed the application of *Bacillus* PC465 enhances the gut microbial structures, promotes the immune status of shrimp which *per se* protected against WSSV. Despite the needs for additional research to explain mechanisms, some researchers proposed immunomodulatory nature of probiotics as an important factor in observed protection against WSSV (Merrifield et al., 2010b).

## Probiotics and parasitic diseases in fish and shellfish

In general, available information about the probiotic control parasite diseases in fish and shellfish was limited. Dietary administration of *Aeromonas sobria* GC2 BA211 for 14 days at rate of 10^8^ and 10^10^ cells g^−1^, respectively, protected rainbow trout against *Ichthyophthirius multifiliis* parasite and reduced the mortalities from 98 to 0%. On the other hand, *Brochothrix thermosphacta* at dose of 10^10^ cells g^−1^ of feed failed to protect rainbow trout against the skin parasite (Pieters et al., 2008). Atira et al. (2012) assessed the inhibition of the growth of the parasitic *Saprolegnia parasitica* A3 on catfish (*Pangasius hypophthalamus*) using *Lactobacillus plantarum* FNCC 226 under *in vivo* and *in vitro* conditions. They concluded the potential of *L. plantarum* for inhibiting *S. parasitica* and therefore suggested as an environment-friendly means of parasite control in catfish aquaculture.

## Concluding remarks and further perspectives

The review of available literature revealed the promising effects of probiotics on disease resistance of fish and shellfish. Therefore, it can be speculated that this environment friendly dietary supplement will receive increasing attention as an alternative for antibiotic in aquaculture. However, this fact should be kept mind that the results of previous researches revealed that the effects of probiotics are species specific. Therefore, optimum probiont, administration dose and dulactobacilli were among the most studied probiotics in shrimps. The studies reviewed here revealed the potential of lactobacilli to help in resolving the issue of diseases in shrimp culture. Given the primary nature of shrimp immune system as well as sensitivity to disease outbreak, development of such effective, environment-friendly means of disease bio-control is of high importance. The results of the mentioned above studies encourage further studies regarding bio-control of parasite in aquaculture using probiotics. However, the exact mode of actions remained to be clarified. Furthermore, despite promising effects obtained regarding probiotics as bio-control against viral and parasitic disease in aquatic animals, there is very limited research available compared with other immunostimulants. Consequently, extensive research should be performed regarding determination of antiviral nature of known probiotics. The last but not the least, present understanding on modes of action of probiotics effects on immune system is very limited and merit further research, especially the molecular mechanisms of the interactions between the probiotic and host.

## Author contributions

SH and ZZ drafted the manuscript. Y-ZS performed the literature collection. AW participated in this review. All authors performed the critical revision of the article and approved the final version for publication.

### Conflict of interest statement

The authors declare that the research was conducted in the absence of any commercial or financial relationships that could be construed as a potential conflict of interest.
